# Bis-cyclometalated iridium(iii) complexes with terpyridine analogues: syntheses, structures, spectroscopy and computational studies†

**DOI:** 10.1039/d1ra07213g

**Published:** 2021-12-13

**Authors:** Haleema Y. Otaif, Samuel J. Adams, Peter N. Horton, Simon J. Coles, Joseph M. Beames, Simon J. A. Pope

**Affiliations:** School of Chemistry, Main Building, Cardiff University Cardiff CF10 3AT UK popesj@cardiff.ac.uk; UK National Crystallographic Service, Chemistry, Faculty of Natural and Environmental Sciences, University of Southampton Highfield Southampton SO17 1BJ England

## Abstract

Two ligands based upon a 2,6-disubstituted pyridine bridge introduce bis-quinoxalinyl units in a fashion that yields analogues to the archetypal terdentate ligand, 2,2′:6′,2′′-terpyridine. The ligands were synthesised from the key intermediate 2,6-bis(bromoacetyl)pyridine: a new, high-yielding route is described for this reagent. Two ligand variants (differentiated by H/Me substituents on the quinoxaline ring) were explored as coordinating moieties for iridium(iii) in the development of luminescent complexes. Computational studies (DFT approaches employing B3LYP, B3LYP/LANL2DZ, and M062X/LANL2DZ levels) were used to investigate the geometric and coordination mode preferences of the new ligands and two possibilities arose from theoretical investigations: [Ir(N^N^N)_2_]^3+^ and [Ir(N^N^C)_2_]^+^, with the former predicted to be more energetically favourable. Upon synthesis and isolation of the Ir(iii) complexes, X-ray crystallographic studies revealed coordination spheres that were cyclometalated, the structures both showing a [Ir(N^N^C)_2_]PF_6_ arrangement. Further spectroscopic characterization *via* NMR confirmed the ligand arrangements in the complexes, and photophysical studies, supported by DFT, showed that a mixture of metal-to-ligand charge transfer (MLCT) and intra-ligand charge transfer (ILCT) character is likely to contribute to the emission features of the complexes, which phosphoresce orange-red (*λ*_em_ = 580–618 nm). The emission wavelength was influenced by the substituents on the quinoxaline ring (H *vs.* Me), thereby implying further tuneability is possible with future ligand iterations.

## Abstract 2

Bis-terdentate Ir(iii) complexes incorporate two cyclometalated N^N^C bis-quinoxalinyl type ligands derived from the condensation of 2,6-bis(bromoacetyl)pyridine and different *o*-phenylenediamines.

## Graphical abstract

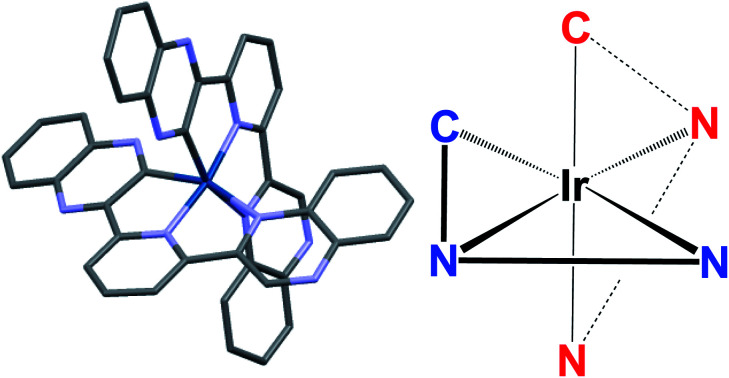

## Introduction

Luminescent coordination compounds based upon organometallic iridium(iii) species have attracted significant attention over the last 25 years.^1^ Interest and continued developments have been driven by the important applications of such compounds. Areas such as photoredox catalysis,^2^ light emitting diodes,^3^ electroluminescent devices^4^ and bioimaging applications^5^ have all continued to benefit from the use of luminescent cyclometalated Ir(iii) complexes. Since the electronic properties of such complexes can be tuned using the coordinating ligands, continued efforts are focused upon new chelating agents that can impart useful physical properties upon the complex.^6^ Homoleptic [Ir(ppy)_3_] (where ppy = 2-phenylpyridine) or cationic [Ir(ppy)_2_(L)]^+^ complexes are the archetypal luminescent Ir(iii) species where the ppy ligand cyclometalates in a bidentate C^N fashion. In fact many ligands developed for cyclometalation with Ir(iii) are often variants on the ppy structure^7^ and thus intrinsically bidentate in nature. In contrast, luminescent Ir(iii) complexes incorporating terdentate ligands are less common, despite initial reports of such systems >25 years ago.

**Scheme 1:**
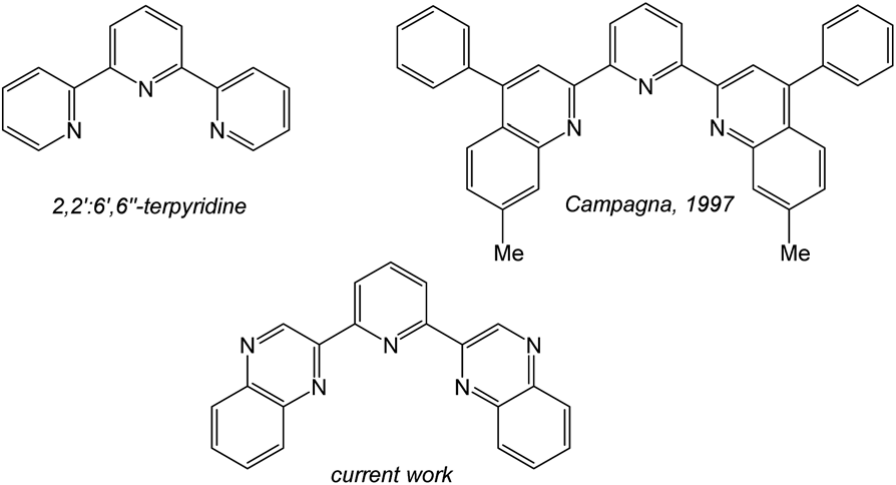
A comparison of terdentate ligand structures directly relevant to this work.

Firstly, [Ir(terpy)_2_]^3+^ (where terpy = 2,2′:6′,2′′-terpyridine) is a well-known compound that possesses a N^N^N binding mode, and shows a structured emission band in the blue-green region attributed to ligand-centred triplet states^8^ (and thus contrasts with the typical ^3^MLCT/^3^LLCT emission character of [Ir(ppy)_3_] and [Ir(ppy)_2_(bipy)]^+^). Heteroleptic luminescent Ir(iii) complexes that incorporate terpy have also been reported to possess ligand tuneable emission characteristics.^9^ Also of direct relevance is the prior work of Campagna (Scheme 1) on the complexation of 2,6-bis(7′-methyl-4′-phenyl-2′-quinolyl)pyridine^10^ where it was shown that the ligand cyclometalates with Ir(iii) to give both [Ir(N^N^C)_2_]^+^ and [Ir(N^N^N)(N^N^C)]^2+^ luminescent species.^11^

Several groups^12–14^ have also reported a number of important Ir(iii) complexes that incorporate structural analogues of terpy, including N^N^C (*via* phenyl-2,2′-bipyridine derivatives), N^C^N (*via* dipyridyl-benzene derivatives) and C^N^C (*via* 2,6-diphenylpyridine) coordination modes at Ir(iii). The design principles and applications of emissive bis-terdentate Ir(iii) complexes have been reviewed^15^ highlighting particularly attractive assemblies for photoactive multi-metallic constructs; terdentate ligands allow the design of linear (or pseudo axial symmetry) and rigid assemblies which are advantageous when studying intramolecular electron or charge transfer phenomena^16^ (commonly mixed ligand formulations support the design of such systems^17^). Terdentate ligands can also be deployed as bridging moieties in dinuclear Ir(iii) complexes, which have shown impressive emission properties,^18^ and have been reported as emissive components of polymers.^19^ However, among these reports there are clearly inherent challenges reported with the purification of the target complexes because of the kinetic inertness of Ir(iii) and the different isomeric forms that often arise from terdentate ligand systems.^15^

**Scheme 2:**
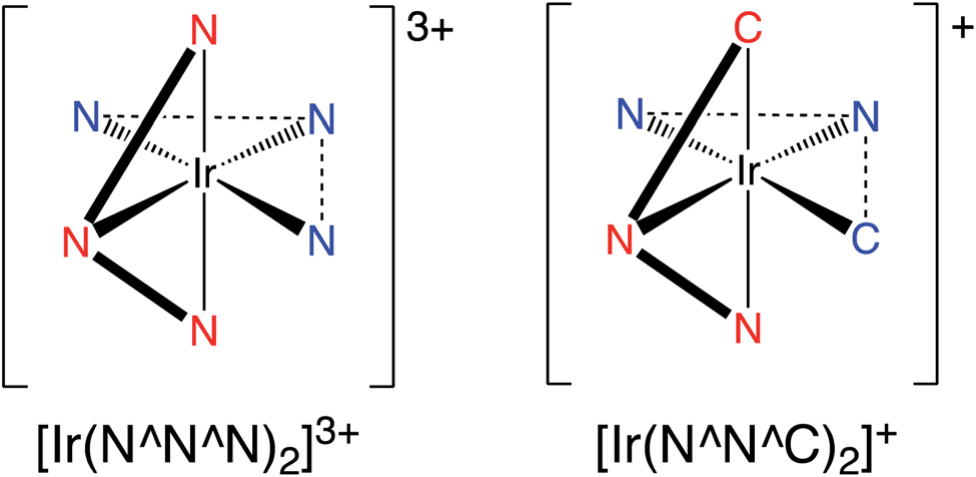
A comparison of the two coordination modes under consideration in a bis-terdentate coordination sphere at Ir(iii).

Inspired by these earlier studies, and as part of our ongoing investigations into quinoxaline-based ligand systems for Ir(iii),^20^ this current work describes the successful synthesis of the first 2,6-bis-quinoxalinyl pyridine analogues of terpyridine. This new ligand architecture provides an extended π-conjugation to the ligand framework and can be synthesised in a manner that advantageously allows a degree of functionalisation at the quinoxaline moiety. Therefore the aim of the current study was to structurally clarify the coordination chemistry preferences (Scheme 2) of the new ligands with Ir(iii), and reveal their resultant spectroscopic properties, including photophysical attributes.

## Results and discussion

### Ligand design and synthesis

**Scheme 3:**
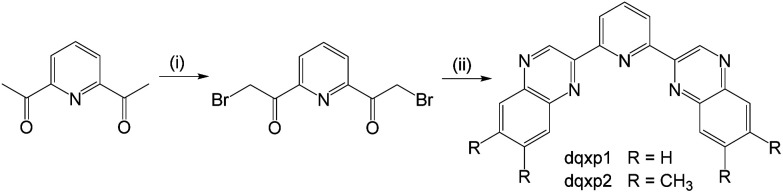
Synthetic pathway to the target ligands. Reagents and conditions: (i) excess 1,4-dioxane dibromide, dioxane, heat, 3 h; (ii) 2 eq. of 1,2-phenylenediamine or 4,5-dimethyl-1,2-phenylenediamine, 1,4-dioxane, heat, 16 h.

The ligands were synthesised (Scheme 3) in two steps from commercially available 2,6-diacetylpyridine. Despite its obvious potential in ligand development, 2,6-bis(bromoacetyl)pyridine has only been reported a handful of times^21^ and the bromination routes from the diacetyl precursor typically use either *N*-bromosuccinimide, HBr and/or Br_2_. However, over-bromination is common with these reagents and thus purification can be tedious and limiting to overall yields; the mixture of compounds from the bromination of a diacetyl presumably explains the relatively poor yields previously reported for this compound. As an alternative, we investigated the use of dioxane-dibromide^22^ as an easily handled, solid form of brominating agent. Heating 2,6-diacetylpyridine in 1,4-dioxane with dioxane-dibromide over 3 h led to the formation of 2,6-bis(bromoacetyl)pyridine, which could be obtained in multi-gram (>10 g) scale, in good yield (67%), and without need for further purification. As indicated by ^1^H NMR analyses, these conditions retard unwanted over-bromination that is commonly associated with other reagents and is thus free of time-consuming purification procedures which can compromise yields. 2,6-Bis(bromoacetyl)pyridine was then reacted with either 1,2-phenylenediamine or 4,5-dimethyl-1,2-phenylenediamine to give the corresponding bis-quinoxalinyl pyridine derivatives dqxp1 and dqxp2, respectively.

Purification of the ligands by column chromatography returned moderate yields, with the products characterized by ^1^H and ^13^C NMR spectroscopy and high resolution mass spectrometry (HRMS). NMR studies of the ligands showed resonances in concordance with a plane of symmetry through the central pyridyl ring, evidenced by a triplet peak at *ca.* 8 ppm for the proton at the 4-position of the pyridine, as well as a doublet with an integral of 2H at 8.60 ppm. A singlet proton resonance was observed *ca.* 10 ppm consistent with the formation of the quinoxaline heterocyclic moiety.

Reaction of the ligands with hydrated IrCl_3_ initially gave the putative intermediate ‘{IrCl_3_(dqxp)}’, which is likely to be a dimeric species, [IrCl(dqxp)]_2_-μ-Cl_2_, as reported by Williams^13^ in previous studies on different terdentate cyclometalating ligands. These intermediate species possessed very limited solubility preventing any meaningful characterisation *via* NMR spectroscopy. The crude intermediate was therefore reacted with another equivalent of the ligand in refluxing ethylene glycol. Precipitation of the crude complexes was achieved as their hexafluorophosphate salts. These materials were then subjected to column chromatography (silica gel; DCM/MeOH solvent gradient), where a number of coloured bands were observed. The dominant orange-red band was collected and drying *in vacuo* afforded the purified species in modest yields.

### Structural characterisation of the complexes: X-ray crystallography studies

Single crystals suitable for diffraction studies were obtained for both complexes using vapour diffusion of diethyl ether into concentrated acetonitrile solutions. Full data collection parameters are provided in the ESI (Table S1†). The structural analyses show the complexes to be [Ir(dqxp1^−^)_2_]PF_6_ (red prism like crystals) and [Ir(dqxp2^−^)_2_]PF_6_ (red plate-shaped crystals). The structures unequivocally reveal that both ligands adopt a N^N^C coordination mode and are anionic and cyclometalated. The metalated carbon is thus adjacent to the nitrogen of one of the quinoxaline rings. This coordination mode imparts a distorted octahedral coordination geometry at Ir(iii). The complex unit is therefore mono-cationic and charge balanced by one hexafluorophosphate counter anion.

Table 1 shows the bond lengths describing the coordination sphere of the complexes. In both structures the mutually *trans* Ir–N_py_ bond lengths are slightly shorter than the Ir–N_qx_ bonds. The structure for [Ir(terpy)_2_](PF_6_)_3_ describes^23^ Ir–N bonds in the range 1.978–2.058 Å and is therefore comparable to the majority of the Ir–N distances reported here. It is evident that both Ir–N and Ir–C bond lengths compare closely to monocationic complexes that feature cyclometalated 2-phenylquinoxaline ligands.^20^ Examination of the bite angles within each coordinated terdentate ligand shows that the three *trans* angles are again comparable to other structures incorporating terpy ligands.^24^ In the two complexes described here, the N–Ir–N *trans* angles are approximately 5° less than the C–Ir–N angles (Table S2, ESI†). We note that the *trans* N–Ir–N bond angle is closer to 180° in the methylated complex, [Ir(dqxp2^−^)_2_]PF_6_.

**Table 1:** Experimental bond lengths obtained from the X-ray crystal structures

[Ir(dqxp1^−^)_2_]PF_6_			[Ir(dqxp2^−^)_2_]PF_6_		
Atom	Atom	Length/Å	Atom	Atom	Length/Å
Ir(1)	N(1)	1.999(2)	Ir(1)	N(1)	2.009(5)
Ir(1)	N(31)	1.997(2)	Ir(1)	N(4)	2.191(5)
Ir(1)	N(2)	2.183(2)	Ir(1)	N(31)	2.017(5)
Ir(1)	N(32)	2.199(2)	Ir(1)	N(34)	2.198(5)
Ir(1)	C(45)	1.993(3)	Ir(1)	C(1)	1.999(6)
Ir(1)	C(15)	1.989(3)	Ir(1)	C(31)	2.005(7)

The packing diagrams for both species are included in the ESI (Fig. S2 and S3†) and reveal long-range ordering induced by intermolecular interactions supported by π-stacking of the aromatic ligand constituents. Interestingly, for [Ir(dqxp1^−^)_2_]PF_6_ it is the N-coordinated quinoxaline rings that stack (at 3.562 Å), whereas for [Ir(dqxp2^−^)_2_]PF_6_ it is the C-coordinated quinoxaline rings that promote stacking (at 3.677 Å).

### Characterisation of the complexes: spectroscopic studies

**Fig. 1:**
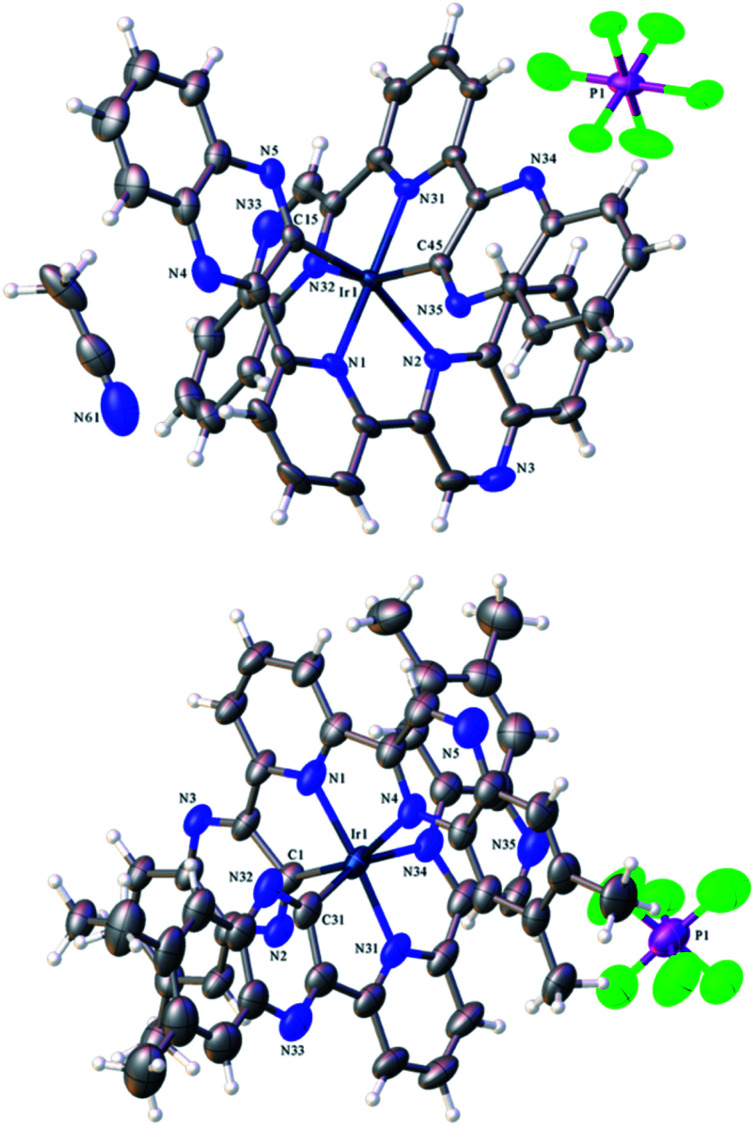
X-ray crystal structure of [Ir(dqxp1^−^)_2_]PF_6_ (top)and [Ir(dqxp2^−^)_2_]PF_6_ (bottom).

As discussed, the X-ray diffraction studies demonstrated that isolated crystals of the Ir(iii) complexes possessed the cyclometalated coordination sphere. ^1^H NMR spectroscopic characterization of the bulk samples again supported the isolation of the [Ir(N^N^C)_2_]^+^ isomeric form of each species. For example, [Ir(dqxp2^−^)_2_]PF_6_, each methyl environment becomes inequivalent and were observed between 1.92–2.23 ppm. Three unique aromatic resonances at 8.54, 8.64 and 9.01 ppm (see Fig. S3, ESI†) were attributed to the loss of symmetry across the bridging pyridyl ring, and a further five quinoxaline-based ^1^H resonances were observed as singlets at 6.47, 7.03, 7.40, 7.71 and 9.88 ppm. The overall loss of one aromatic proton environment *versus*dqxp2 is due to metalation at the 3-position of one of the quinoxaline moieties. The spectra confirm the asymmetry of the ligand(s) and the inequivalence of the quinoxaline moieties induced by the N^N^C chelating mode. The coordination geometry of [Ir(dqxp2^−^)_2_]PF_6_ positions the proton at the 8 position of the quinoxaline ring above the plane of the pyridine ring (Fig. 1; note the larger N–Ir–N *trans* angle may subtly promote this interaction), which may explain the upfield shift to 6.47 ppm.

HRMS was obtained for both complexes and showed the correct mass, and corresponding isotope pattern, for the loss of one PF_6_^−^ counter ion thus implying a mono-cationic [Ir(dqxp^−^)_2_]^+^ fragment.

### Prediction of structures and electronic properties using computational studies

An initial investigation using computational DFT approaches compared the two coordination modes that were deemed feasible for the ligand–metal combination. Thus, two hypothetical structures (using dqxp1) were calculated that compared the relative energies of the [Ir(N^N^N)_2_]^3+^ {*cf.* [Ir(terpy)_2_]^3+^} and [Ir(N^N^C)_2_]^+^ coordination isomers (Fig. S4, ESI†). The data for each functional used (B3LYP/SDD, B3LYP/LANL2DZ, M062X/LANL2DZ) is tabulated in the ESI (Tables S3–S5†). In summary, the calculated energies are similar for both isomers, but for each functional deployed it was noted that the [Ir(N^N^N)_2_]^3+^ structure was predicted to be more energetically favourable.

The experimental X-ray diffraction determination of the structural characteristics of both Ir(iii) complexes provided the ideal benchmark against which to verify the density functional theory (DFT) approaches used to help describe the electronic properties of the two complexes. The geometry optimization of the two complexes were carried out for both their lowest singlet and triplet states at the DFT (B3LYP/SDD,6-31G* level of theory), with an implicit CHCl_3_ solvent (IEFPCM). All optimisations were confirmed as minima through harmonic vibrational frequency calculations.

**Fig. 2:**
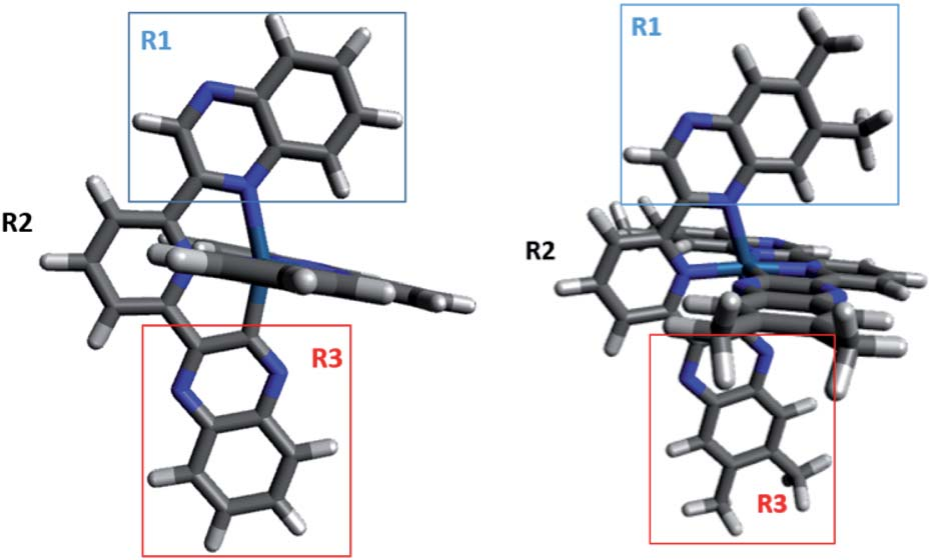
Optimized geometries of [Ir(dqxp1^−^)_2_]PF_6_ (left) and [Ir(dqxp2^−^)_2_]PF_6_ (right). The two complexes have three moieties highlighted, each of which is used to provide a more effective molecular orbital decomposition of the system (R1 = *N*-quinoxaline; R2 = pyridine; R3 = C-quinoxaline).

The optimized geometries of both complexes obtained by DFT are shown in Fig. 2. The calculated bond lengths for the studied complexes are in good agreement with their corresponding X-ray crystallographic values, with an average deviation of less than 0.05 Å (Table S6, ESI†). Typically, the optimised bond lengths were slightly longer in comparison with the experimental data, which might be because the DFT calculations were carried out in implicit solvent while the experimental data were recorded in solid state.

**Fig. 3:**
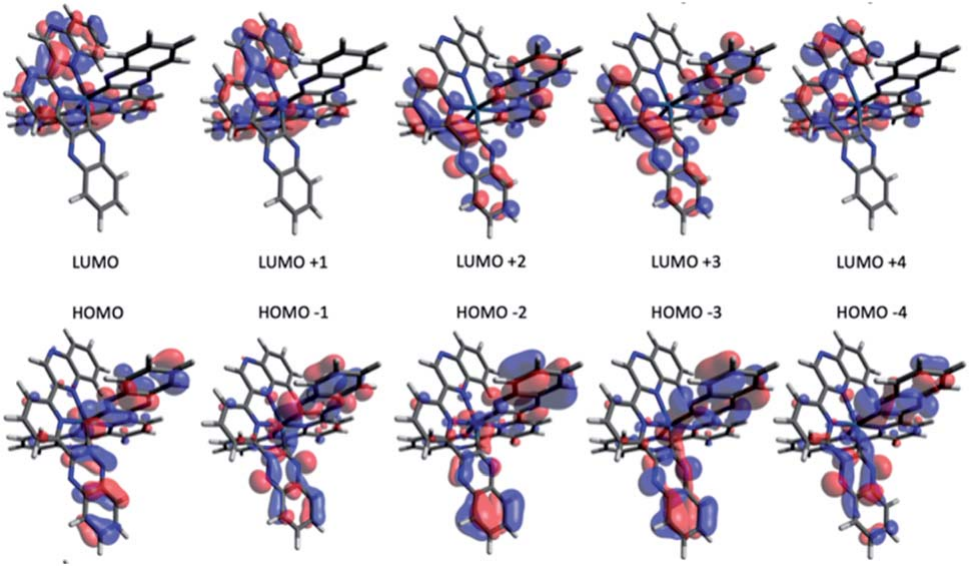
Renderings of the frontier molecular orbitals of the [Ir(dqxp1^−^)_2_]PF_6_ complex.

To investigate the effect of terdentate ligands on the electronic structures, molecular orbital decomposition analyses were performed on both complexes using the GaussSum software package. Each ligand was divided into three separate moieties, the C-quinoxaline (R3), *N*-quinoxaline (R1) and pyridinyl groups (R2), with the iridium metal core constituting its own group. This enabled a more in-depth examination of the complex contributions when compared to treating each whole ligand as an individual group. The results of the decomposition analysis for the frontier orbitals of the singlet ground state for both complexes with each of the ligand groups labelled are shown in Tables 2 and 3; renderings of the frontier molecular orbitals of the [Ir(dqxp1^−^)_2_]PF_6_ complex are shown in Fig. 3 (the equivalent information for [Ir(dqxp2^−^)_2_]PF_6_ is shown in Tables S7 and S8, ESI†).

**Table 2:** The decomposition analysis of the singlet ground state frontier orbitals of [Ir(dqxp1^**−**^)_2_]^+^

Orbital	Moiety contribution to orbital (%)						
	Ir 5d	Q1			Q2		
		R1	R2	R3	R1	R2	R3
LUMO+4	1	33	15	0	34	15	0
LUMO+3	1	0	21	28	0	21	28
LUMO+2	1	1	19	29	1	20	29
LUMO+1	4	38	11	0	35	10	0
LUMO	4	34	12	1	37	13	1
HOMO	29	2	3	30	2	3	30
HOMO−1	8	3	3	38	3	4	40
HOMO−2	10	2	5	40	2	4	37
HOMO−3	6	1	3	42	1	4	43
HOMO−4	7	3	4	39	3	4	39

**Table 3:** Computed values for the absorption and emission maxima of the two Ir(iii) complexes

Complex	S_0_ → T_*n*_ Abs. (nm)	Vertical T_1_ → S_0_ Em. (nm)	Adiabatic T_1_ → S_0_ Em. (nm)
[Ir(dqxp1^−^)_2_]^+^	513	698	581
[Ir(dqxp2^−^)_2_]^+^	527	689	587

Consistent with previously reported 2-phenylquinoxaline Ir(iii) complexes,^20^ the HOMO in both complexes is constructed principally of the contributions from the Ir d-orbital and π-orbitals of both ligands, located predominantly on the C-quinoxaline moieties. This is in contrast with the LUMO, localized largely on the *N*-quinoxaline moieties, with a contribution from pyridyl moieties. It is noteworthy (from Tables 2 and S7†) that the two ligands in the [Ir(dqxp^−^)_2_]PF_6_ complexes are predicted to be functionally degenerate, with contribution values falling within a few percentage points of each other, where they differ at all, for any given orbital.

TD-DFT using CAM-B3LYP functional and stationary point calculations were carried out on the two complexes with the aim of computing and analysing the experimentally observed absorption and emission bands. The vertical and adiabatic spin-forbidden transitions of the complexes were estimated, based on the optimized singlet ground state and triplet excited-state geometries, and the results are shown in Table 3. The first five singlet-to-singlet transitions of the complexes derived from the singlet geometry are reported below in Tables 4 and S8.†

**Table 4:** The wavelength, oscillator strengths and molecular orbital contributions for the first five singlet-to-singlet excited states for [Ir(dqxp1^**−**^)_2_]PF_6_. Molecular orbital contributions less than 10% have been omitted for clarity

	Transition	Contributing MOs
1	383.91 nm, *f* = 0.0099	HOMO → LUMO (77%)
2	383.64 nm, *f* = 0.0179	HOMO → LUMO+1 (75%)
3	354 nm, *f* = 0.0011	HOMO−5 → LUMO+3 (29%)
		HOMO−4 → LUMO+2 (47%)
4	352.64 nm, *f* = 0.0007	HOMO−5 → LUMO+2 (36%)
		HOMO−4 → LUMO+3 (40%)
5	350.12 nm, *f* = 0.0067	HOMO−7 → LUMO+1 (12%)
		HOMO−6 → LUMO (40%)

The calculated spin-forbidden vertical absorption transitions fall in a similar region as previously studied 2-phenylquinoxaline Ir(iii) complexes,^20^ suggesting that the shoulder peak observed at the longer wavelengths (> 425 nm) in the experimental UV-vis absorption spectra are due to direct photoexcitation to lower lying triplet state(s). Similarly, the vertical and adiabatic emission estimations are a good match for experiment, with predicted wavelengths occurring within the same region as the complex emission spectra, supporting the assignment of the long-lived emission feature being spin-forbidden in nature. The calculated adiabatic band positions provide a reasonable quantitative match for the experimentally determined values.

The lowest energy transitions expected from the spin-allowed component of the UV-vis absorption spectra extend only as far as 380–390 nm, in keeping with the assignment of spin-forbidden vertical transitions accounting for the longer wavelength absorption bands (400 nm < *λ* < 500 nm). The predicted magnitude of spin-forbidden absorption appears to be more significant in these tridentate complexes than in related 2-phenylquinoxaline Ir(iii) complexes,^20^ given the relatively high intensity of absorption beyond the wavelengths that are ascribed to singlet–singlet processes. Combinations of these results with the decomposition analysis show that the first and second transitions are composed of charge transfer from the Ir centre and R3 ligand component, to the R1 and R2 moieties, which can thus be described in terms of mixed MLCT/ILCT/LLCT character. The low oscillator strengths of these early transitions are likely due to the minimal wavefunction overlap expected between the R3 moiety and the R1 and R2 moieties due to their spatial separation.

### Photophysical properties

Firstly, the UV-vis absorption spectra of the ligands were obtained in EtOH (see Fig. S5, ESI†) and show two primary features: an intense sharp feature around 260 nm and a weaker, broader band with a hint of vibronic coupling at 300–375 nm. Both bands are slightly bathochromically shifted for dqxp2. In both cases these bands are attributed to various spin-allowed, intra-ligand transitions that are likely to be π → π* in nature.

**Fig. 4:**
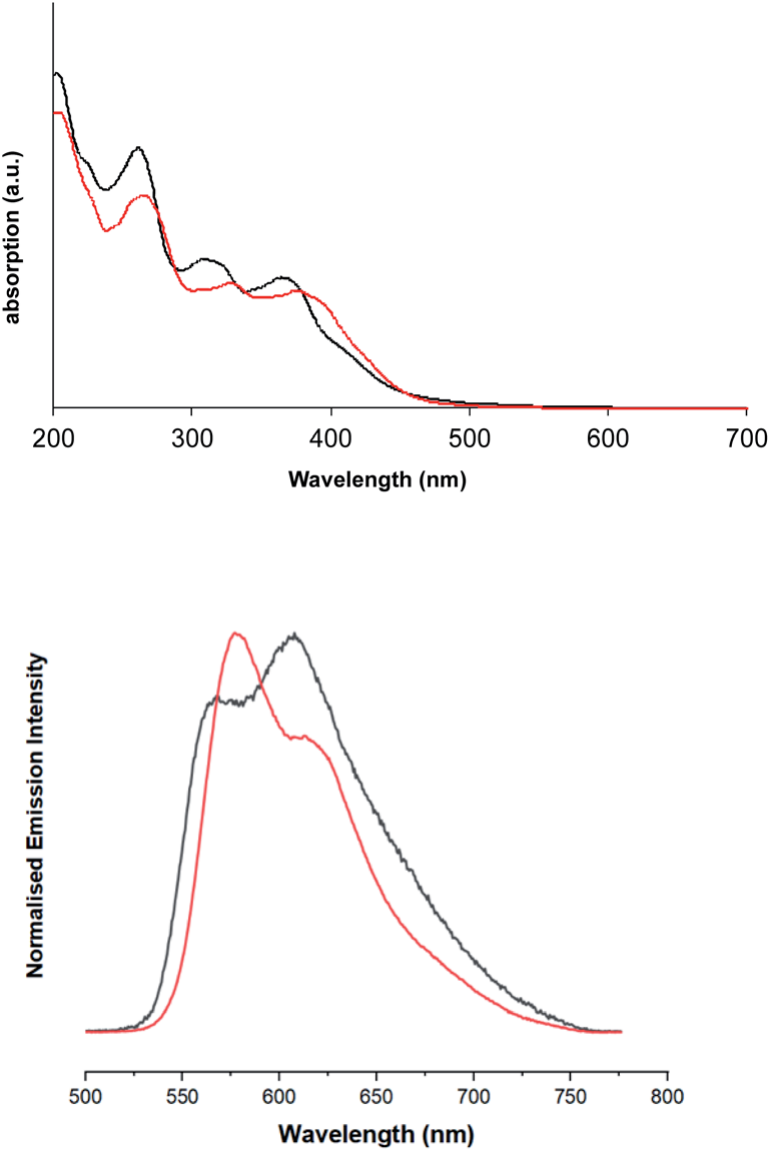
Solution state UV-vis absorption (top) and emission (bottom) spectra of [Ir(dqxp1^−^)_2_]PF_6_ complex (black), and [Ir(dqxp2^−^)_2_]PF_6_ complex (red).

In comparison the spectra of the complexes (Fig. 4), suggest that the absorption bands 300 nm < *λ* < 370 nm can be ascribed to ligand centred transitions, with some contribution likely from metal-to-ligand charge transfer (MLCT) transitions. Consistent with prior literature, the feature between 350 nm < *λ* < 420 nm can be tentatively assigned as arising from spin-allowed ^1^MLCT/^1^ILCT/^1^LLCT transitions, as supported by DFT. Both complexes show a prominent feature in the region 350 nm < *λ* < 400 nm, which has a shoulder feature extending to approximately 550 nm. This shoulder contains a visible low-intensity feature in both cases, that is ascribed to spin-forbidden singlet–triplet absorptions, likely ^3^MLCT in nature, as discussed earlier in the DFT investigation. As with the free ligands, the effect of methylation on the quinoxaline backbone is to bathochromically shift all the main absorption features of [Ir(dqxp2^−^)_2_]PF_6_.

**Fig. 5:**
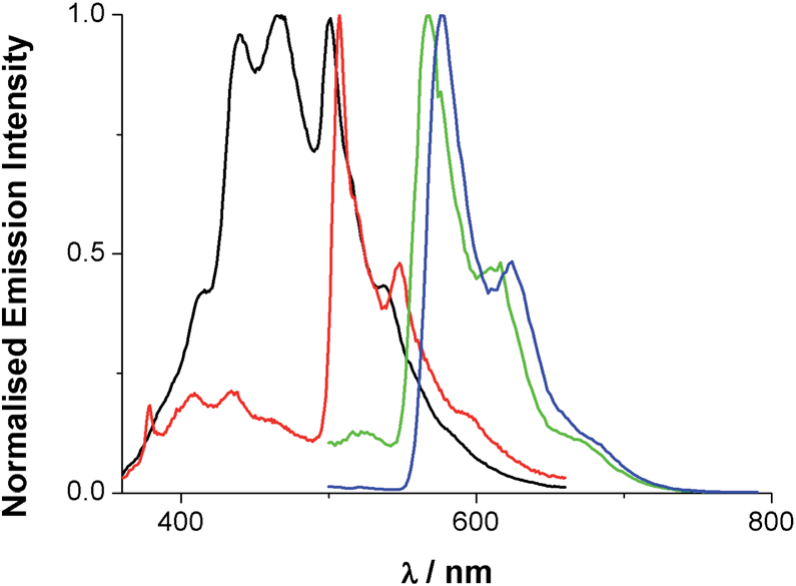
Total emission spectra of dqxp1 (black), dqxp2 (red), [Ir(dqxp1^−^)_2_]PF_6_ (green) and [Ir(dqxp2^−^)_2_]PF_6_ (blue) in 3 : 1 EtOH : MeOH glass at 77 K [*λ*_exc_ = 340 (ligand), 400 nm (complex)].

Upon excitation at 355 nm, the emission properties (aerated CHCl_3_) of the complexes (Fig. 4 and Table 5) display a broad luminescence feature around 580–615 nm. In both cases this peak possesses some vibronic structure. The relative intensities of the two main features of the peak are inverted when the methyl substituents are present. Total emission spectra for both complexes were also recorded (Fig. 5) at 77 K in a solvent glass (3 : 1 EtOH : MeOH) and show much more pronounced resolution of the vibronic progression. Under identical conditions, the free ligands display vibronically structured emission features consistent with an intra-ligand ^3^(π–π*) emitting state.

**Table 5:** Photophysical properties of the Ir(iii) complexes^a^

Complex^a^	*λ*_abs_ (*ε* × 10^4^/M^−1^ cm^−1^)/nm	*λ*_em_ b/nm	*τ*_obs_ b/ns	*ϕ* c (%)
[Ir(dqxp1^−^)_2_]PF_6_	267(9.0), 318 sh (5.2), 367 (4.5)	610	410	3
[Ir(dqxp2^−^)_2_]PF_6_	273(7.1), 334 (4.2), 378 (3.9)	580	662	3

a In aerated chloroform.

b Excitation at 355 nm.

c Using [Ru(bipy)_3_](PF_6_)_2_ as standard (1.6% in aerated MeCN).^25^

A comparison of the spectra for dqxp2 and [Ir(dqxp2^−^)_2_]PF_6_ clearly shows a close spectral resemblance to the vibronic progression, which presumably belongs to the quinoxaline-centred vibrational modes, but one which is accompanied by a significant bathochromic shift upon coordination to Ir(iii). Interestingly, the appearance of the room temperature emission band was also sensitive to concentration; increasing the concentration of the complex resulted in a general bathochromic shift and a broadening of the peak (Fig. S6, ESI†). Solvatochromic studies were also undertaken revealing a small variance in emission properties as a function of solvent polarity (see Tables S9 and S10, ESI†). This confirms that although MLCT character is likely to contribute the transition is mainly ligand-based and may not result in a strong change in dipole moment. In aerated solution [Ir(dqxp1^−^)_2_]PF_6_ and [Ir(dqxp2^−^)_2_]PF_6_ displayed observed lifetimes of 410 and 662 ns, respectively. These lifetimes were extended to around 1 microsecond under deoxygenated conditions, consistent with a phosphorescent emitting state.

It is pertinent to compare these data with related bis-cyclometalated complexes of cationic Ir(iii) with 2-phenylquinoxaline (2-pqx) ligands, [Ir(2-pqx)_2_(bipy)]^+^, recorded under similar conditions.^20*b*^ The emission maxima and lifetimes are broadly comparable between the two classes of compound, however the luminescence spectral profiles are slightly different: the [Ir(2-pqx)_2_(bipy)]^+^ species possess broad featureless emission profiles attributed to ^3^MLCT/^3^ILCT character (the LUMO is predicted to be based upon the quinoxaline moiety not the bipy ligand). Despite comparable spectral data, the predicted HOMO and LUMO contributions are subtly different for the archetypal bis-cyclometalated cationic complex [Ir(ppy)(bipy)]^+^, which also shows a broad featureless emission profile (*λ*_em_ 602 nm, *ϕ*_em_ 9.3%, *τ*_obs_ 275 ns in aerated MeCN).^7^ The HOMO of [Ir(ppy)(bipy)]^+^ has both Ir and phenyl contributions and the LUMO is localised solely on the ancillary bipy ligand resulting in a predicted ^3^MLCT/^3^LLCT mixture to the emissive character. As discussed earlier, [Ir(terpy)_2_]^3+^ is therefore different, with a highly structured blue-green phosphorescence emission band which is attributed to a ligand-centred triplet excited state.

### Time-resolved transient absorption spectroscopy

**Fig. 6:**
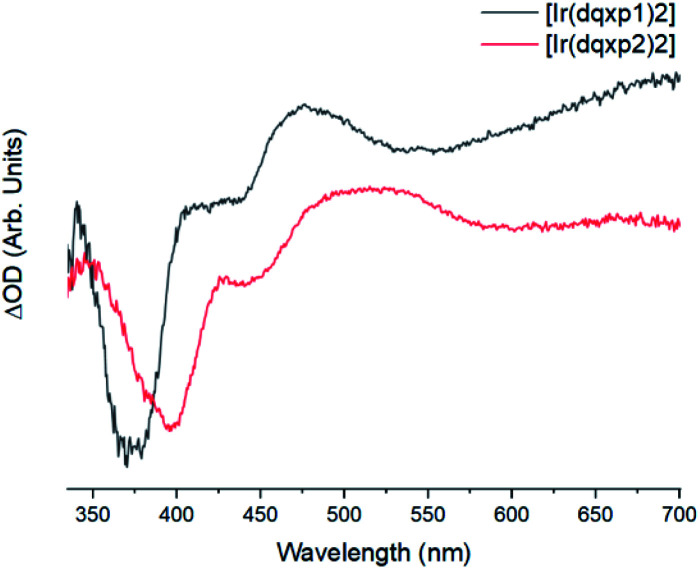
Background and fluorescence subtracted transient absorption spectra of the two complexes in chloroform, *λ*_exc_ = 355 nm, with [Ir(dqxp1^−^)_2_]PF_6_ (black) and [Ir(dqxp2^−^)_2_]PF_6_ (red).

The two complexes present similar transient absorption spectra, shown in Fig. 6, with a set of features that compare to our previous reports on related 2-phenylquinoxaline complexes of Ir(iii).^20^ Both complexes exhibit a strong ground state bleach 350 nm < *λ* < 425 nm, showing good onset and peak maximum agreement with their respective absorption spectra.

**Fig. 7:**
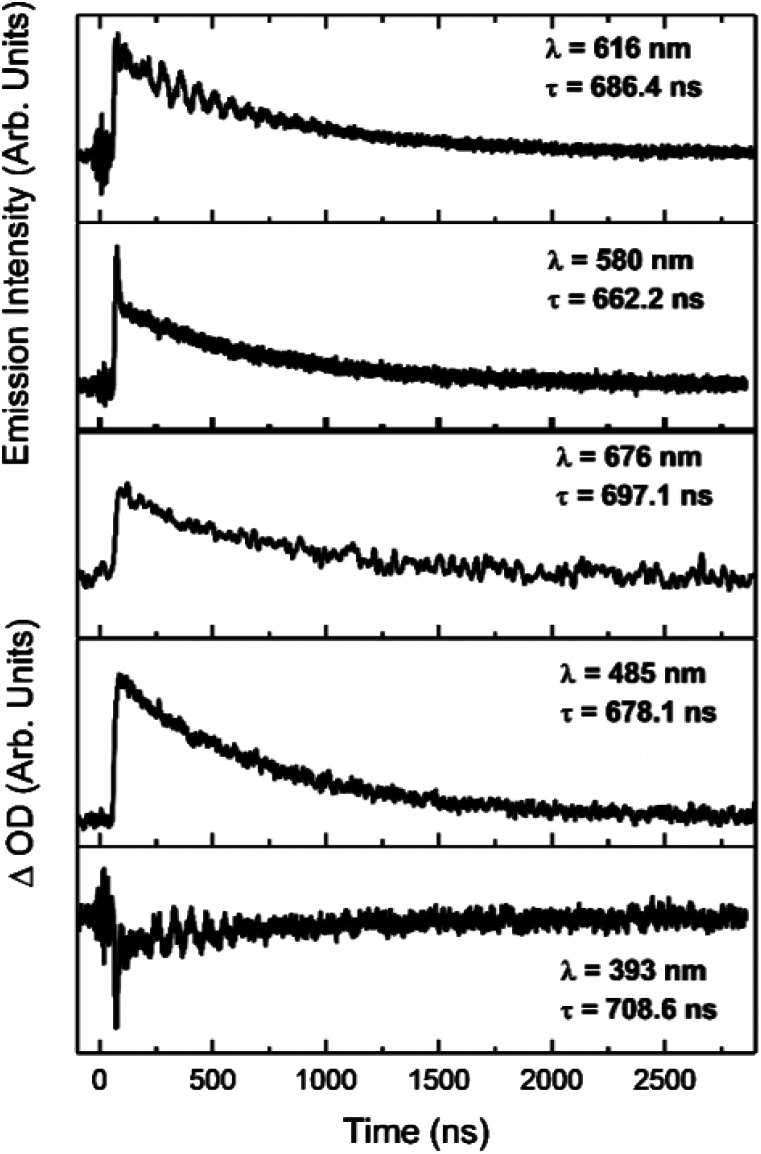
Kinetic traces of the major features of the transient spectra of [Ir(dqxp2^−^)_2_]PF_6_ in chloroform at room temperature, aerated. Wavelengths and lifetimes of each trace are inset.

The increases in optical density observed 450 nm < *λ* < 525 nm in the [Ir(dqxp1^−^)_2_]PF_6_ complex and 475 nm < *λ* < 600 nm in the [Ir(dqxp2^−^)_2_]PF_6_ complex are both assigned to triplet-to-triplet excited state absorption (ESA) bands. These absorption bands are likely predominantly MLCT and ILCT in origin. Both complexes also display a broad, structureless increase in optical density in the longer wavelength region of their spectra, significantly more pronounced in the case of the [Ir(dqxp1^−^)]PF_6_ complex. Similarly, both are assigned to triplet-to-triplet ESA, perhaps arising from transitions from T_*n*>1_. Each feature, including the ground state bleach and the long wavelength ESA bands exhibit similar lifetimes (Fig. 7) suggesting that all the features correlate with the same photoexcitation, ISC process and subsequent deactivation.

## Conclusions

We have reported two new 2,6-bis-quinoxalinylpyridine terdentate ligands that should be considered as structural analogues of terpyridine. Working towards these ligands identified dioxane-dibromide as an excellent and selective brominating agent to yield the key intermediate, 2,6-bis(bromoacetyl)pyridine, in multigram scale. The ready availability of this intermediate potentially provides an important building block to other new multidentate ligands that should find important uses in coordination chemistry and its myriad applications. Upon coordination with Ir(iii), structural and spectroscopic studies clarified that the ligands coordinate in a terdentate manner, *via* cyclometalation, yielding mono-cationic complexes. This renders the ligands in an unsymmetrical arrangement at Ir(iii), giving both C-coordinated and N-coordinated quinoxaline donors. The site of Ir–C bond formation is of interest as it is in the 3-position of the quinoxaline ring and thus adjacent to one of the ring nitrogen atoms. We noted that supporting computational DFT studies predicted that there was no energetic preference for the N^N^C mode over the N^N^N {*cf.* [Ir(terpy)_2_](PF_6_)_3_} coordination mode.

The isolated Ir(iii) complexes are photoluminescent, displaying moderately structured emission in the orange-red region of the spectrum, which was characterized as a phosphorescence of ^3^MLCT/^3^ILCT character. Supporting DFT studies suggest that the HOMO likely comprises contributions from the Ir 5d orbitals and the C-coordinated quinoxaline moiety; the LUMO is likely to be dominated by the N-coordinated quinoxaline unit. Thus, functionalisation of the bis-quinoxalinyl units provides some opportunity for tuning the photophysical attributes of these species.

Of course, given the breadth and importance of coordination chemistry demonstrated by 2,2′:6′,2′′-terpyridine(s), the new bis-quinoxalinyl analogues described herein clearly merit further investigation with a range of different metal ions.

## Experimental

All reactions were performed with the use of vacuum line and Schlenk techniques. Reagents were commercial grade and were used without further purification. ^1^H and ^13^C{^1^H} NMR spectra were obtained on a Bruker Avance dpx 400 spectrometer, and were recorded in CDCl_3_, or CD_3_CN solutions. ^1^H, ^13^C{^1^H} chemical shifts (*δ*) were determined relative to internal tetramethylsilane, Si(CH_3_)_4_ and are given in ppm. Low-resolution mass spectra were obtained by the staff at Cardiff University. High-resolution mass spectra were carried out by the staff at Cardiff University and the EPSRC National Mass Spectrometry Service at Swansea University, UK. Photophysical data was obtained on a Jobin Yvon-Horiba Fluorolog-3 spectrometer fitted with a JY TBX picosecond photodetection module in CHCl_3_The pulsed source was a 355 nm output. Luminescence lifetime profiles were obtained using the Jobin Yvon – Horiba FluoroHub single photon counting module and the data fits yielded the lifetime values using the provided DAS6 deconvolution software. Quantum yields using [Ru(bipy)_3_](PF_6_)_2_ as standard (1.6% in aerated MeCN)^25^ and the following equation:
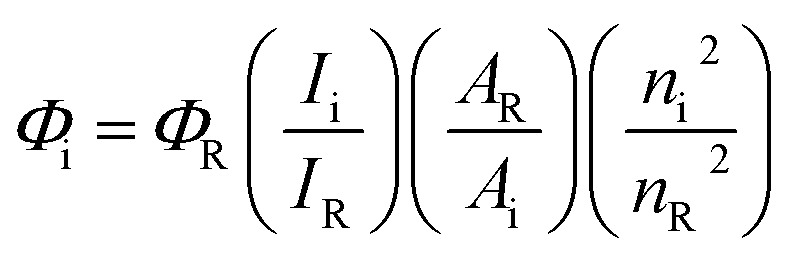


IR spectra were recorded on an ATR equipped Shimadzu IRAffinity-1 spectrophotometer. UV-vis data were recorded as solutions on a PerkinElmer Lamda 20 spectrophotometer.

### X-ray crystallography

#### Data collection and processing

Suitable crystals of [Ir(dqxp1^−^)_2_]PF_6_ and [Ir(dqxp2^−^)_2_]PF_6_ were selected and data collected following a standard method.^26^ In each case, a crystal was selected and mounted on a MITIGEN holder in perfluoroether oil on either a Rigaku FRE+ diffractometer equipped with VHF Varimax confocal mirrors and an AFC12 goniometer and HyPix 6000 detector (for [Ir(dqxp1^−^)_2_]PF_6_) or a Rigaku 007HF diffractometer equipped with Varimax confocal mirrors and an AFC11 goniometer and HyPix 6000 detector (for [Ir(dqxp1^−^)_2_]PF_6_). The crystals were kept at a steady *T* = 100(2) K during data collection using an Oxford Cryosystems low-temperature device. The structures were solved with the ShelXT^27^ structure solution program using the intrinsic phasing solution method and by using Olex2 (ref. 28) as the graphical interface. The models were refined with version 2018/3 of ShelXL^29^ using Least squares minimisation. CCDC 2101342 and 2101343 contains ESI X-ray crystallographic data for [Ir(dqxp1^−^)_2_]PF_6_ and [Ir(dqxp2^−^)_2_]PF_6_ respectively.†

#### Transient absorption measurements

Transient absorption measurements were carried out using an Edinburgh instruments LP920 spectrometer. All spectra were collected using a pump wavelength of 355 nm (third harmonic of a Continuum Surelite II Nd:YAG laser system). The probe light for these measurements was a xenon lamp, affording spectral generation between 300 < *λ* < 800 nm. Wavelength dependent spectra were recorded with a 2.05 nm spectral resolution, collected using an Andor ICCD camera, and integrated over the first 500 ns after the pump laser pulse. The spectra are presented as ΔOD_Xe lamp_, which is simply referred to as ΔOD. Lifetime data was generated using a photomultiplier to collect time resolved signals, with the bandwidth of these data being identical to the camera resolution (2.05 nm). The lifetime data is fit using the Origin 2017 software package, and each data set is fit using a monoexponential function, with no evidence of multiexponential components. Uncertainties in lifetimes are taken from the least-squares fitting algorithm, and are not indicative of the uncertainties in multiple fits or data sets.

### Computational methods

Electronic structure calculations were all performed using density fitted-density functional theory within the Gaussian 09 computational chemistry suite.^30^ All calculations were performed using the Stuttgart–Dresden (SDD) effective core potential and basis set in the treatment of the iridium, in combination with a 6-31G* basis set for all other light atoms. Full geometry optimizations were performed for the cationic complexes utilizing the self-consistent reaction field model (SCRF) which treats the solvent implicitly as a dielectric continuum. In all cases the solvent chosen was chloroform, consistent with that utilized in the majority of the spectroscopic measurements. Chloroform is characterized by an electrical permittivity of *ε* = 4.7113 within the calculations. This computational method models the solvent as surrounding a cavity in which the solute resides, and this cavity is characterized using an integral equation formalism for the polarizable continuum model (IEFPCM). This model represents the system in equilibrium during, for example, an optimization routine: in all excited state calculations a non-equilibrium solvent model is used.

All geometry optimizations were performed using an ultrafine grid and very tight convergence criteria, and the minima were confirmed as stationary points through the computation of harmonic vibrational frequencies, each of which showed no imaginary components. These stationary points were used in single point TD-DFT calculations to compute vertical excitation energies. All TD-DFT calculations were undertaken using a linear response approach. All TD-DFT calculations were also performed with a long range corrected hybrid functional (CAM-B3LYP).

Phosphorescence and spin-forbidden absorption bands were investigated using unrestricted density functional theory to compute parameters associated with the first triplet state (T_1_), using an identical methodology as for the singlet states. Decomposition of the molecular orbital character was performed using the GaussSum software package. Crystal structure overlays with optimised computational structures has been performed using the Chimera software package, which has also been used to calculate root mean squared deviation (RMSD) values for these comparative structures.^31^

### Synthesis

#### Synthesis of 2,6-bis(bromoacetyl)pyridine

To a solution of 2,6-diacetylpyridine (5.0 g, 30.6 mmol) in dioxane (20 mL) heated to 60 °C was added dropwise a suspension of dioxane dibromide (17.0 g, 68.6 mmol) in dioxane (40 mL) over a period of 1 hour. The mixture was maintained at 60 °C throughout and further stirred at this temperature for 2 hours, upon which the solution was allowed to cool to room temperature. The solvent was removed *in vacuo*, and ethanol (15 mL) was added, with the resultant precipitate filtered and washed with ethanol (10 mL) to yield 2,6-bis(bromoacetyl)pyridine (10.6 g, 20.6 mmol, 67%) as a white crystalline solid. ^1^H NMR (400 MHz, CDCl_3_) *δ*_H_ 8.24 (2H, d, *J* = 7.8, NCC*H*CH), 8.06 (1H, t, *J* = 7.8, NCCHC*H*), 4.78 (4H, s, COC*H*_2_Br) ppm; data agrees with previously published results.^21*b*^

#### Synthesis of dqxp1

To a solution of 2,6-bis(bromoacetyl)pyridine (1.0 g, 3.1 mmol) in dioxane (30 mL) was added *o*-phenylenediamine (0.84 g, 7.8 mmol) and the mixture heated to reflux for 16 hours. The reaction was cooled to room temperature, the solvent removed *in vacuo* and the product purified by column chromatography on silica eluting with DCM and MeOH (99 : 1 and 95 : 5) to yield dqxp1 as a grey powder (0.22 g, 0.7 mmol, 21%). ^1^H NMR (400 MHz, CDCl_3_) *δ*_H_ 10.04 (2H, s), 8.60 (2H, d, *J* = 7.9 Hz), 8.03–8.12 (4H, m), 7.99 (1H, t, *J* = 7.9 Hz), 7.67–7.72 (4H, m) ppm. ^13^C{^1^H} NMR (101 MHz, CDCl_3_) *δ*_C_ 154.2, 149.8, 144.2, 142.7, 141.8, 138.4, 130.3, 130.3, 129.7, 129.4, 122.9 ppm. HRMS *m*/*z* found 336.1247; calcd 336.1244 for [C_21_H_14_N_5_]^+^. UV-vis *λ*_max_ (EtOH)/nm (*ε*/dm^3^ mol^−1^ cm^−1^) 213 (20 400), 254 (53 100), 325 sh (20 400), 334 (21 700).

#### Synthesis of dqxp2

4,5-Dimethyl-1,2-diaminobenzene (0.64 g, 4.7 mmol) was added to a solution of 2,6-bis(bromoacetyl)pyridine (0.5 g, 1.6 mmol) in dioxane (15 mL) and treated as above to yield dqxp2 as a grey powder (0.44 g, 1.1 mmol, 72%). ^1^H NMR (400 MHz, CDCl_3_) *δ*_H_ 10.01 (2H, s), 8.69 (2H, d, *J* = 8.0 Hz), 8.10 (1H, t, *J* = 8.0 Hz), 7.95 (4H, overlapping s), 2.56 (12H, overlapping s, Me) ppm. ^13^C{^1^H} NMR (101 MHz, CDCl_3_) *δ*_C_ 154.2, 149.1, 142.9, 141.3, 141.3, 141.1, 140.7, 138.3, 128.6, 128.0, 122.4, 20.4 (*C*H_3_), 20.3 (*C*H_3_) ppm. HRMS *m*/*z* found 392.1870; calcd 392.1870 for [C_25_H_22_N_5_]^+^. UV-vis *λ*_max_ (EtOH)/nm (*ε*/dm^3^ mol^−1^ cm^−1^) 218 (17 300), 258 (27 900), 343 (13 800), 355 sh (11 800).

#### Synthesis of the iridium complexes

dqxp1 (50 mg, 0.15 mmol) and IrCl_3_·*x*H_2_O (44 mg, 0.15 mmol) were suspended in 2-ethoxyethanol (10 mL) and heated to reflux for 2 hours, after which time the reaction was allowed to cool to room temperature and the resulting precipitate filtered and washed with ethanol and diethyl ether to yield ‘[Ir(dqxp1)Cl_3_]’ (69 mg, 0.11 mmol, 73%) which was used without further purification.

‘[Ir(dqxp2)Cl_3_]’ was synthesised in an analogous fashion from dqxp2 (71 mg, 0.18 mmol) and IrCl_3_·*x*H_2_O (54 mg, 0.18 mmol) to yield the title compound as a dark beige solid (94 mg, 0.14 mmol, 75%).

#### Synthesis of [Ir(dqxp1^−^)_2_]PF_6_

‘[Ir(dqxp1)Cl_3_]’ (50 mg, 0.08 mmol) and a further equivalent of dqxp1 (26 mg, 0.08 mmol) were suspended in ethylene glycol (3 mL) and the flask was evacuated and refilled three times with nitrogen. The reaction mixture was heated to reflux in the dark for 2 hours. The mixture was allowed to cool to room temperature and water (20 mL) was added, heated to 60–70 °C and saturated aqueous NH_4_PF_6_ was added and cooled. The precipitate was filtered, washed with water and diethyl ether and dried in air. The product was purified by column chromatography on silica eluting with DCM and MeOH (95 : 5 followed by 9 : 1) and collecting the red band to yield [Ir(dqxp1^−^)_2_]PF_6_ as a red solid (16 mg, 0.02 mmol, 20%). ^1^H NMR (400 MHz, CDCl_3_) *δ*_H_ 9.99 (1H, s), 9.17 (1H, dd, *J* = 1.0, 8.3 Hz), 8.69 (1H, dd, *J* = 0.9, 7.9 Hz), 8.56 (1H, app. t, *J* = 7.9 Hz), 7.66–7.71 (1H, m), 7.64 (1H, dd, *J* = 1.3, 8.2 Hz), 7.36–7.47 (2H, m), 7.31–7.33 (3H, m), 7.61 (1H, dd, *J* = 1.1, 8.5 Hz) ppm. ^13^C{^1^H} NMR (101 MHz, CDCl_3_) *δ*_C_ 159.5, 158.7, 154.2, 150.7, 144.2, 143.9, 143.7, 141.0, 140.2, 139.9, 134.6, 133.1, 130.7, 130.4, 129.7, 128.4, 127.8, 126.4, 125.3, 124.1 ppm. HRMS *m*/*z* found 859.1785; calcd 859.1786 for [C_42_H_24_IrN_10_]^+^. UV-vis *λ*_max_ (MeCN)/nm (*ε*/dm^3^ mol^−1^ cm^−1^) 267 (90 000), 318 (52 000), 367 (45 000).

#### Synthesis of [Ir(dqxp2^−^)_2_]PF_6_

‘[Ir(dqxp2)Cl_3_]’ (70 mg, 0.10 mmol) and dqxp2 (40 mg, 0.10 mmol) were treated as above to yield [Ir(dqxp2^−^)_2_]PF_6_ as a red solid (32 mg, 0.03 mmol, 28%). ^1^H NMR (400 MHz, CD_3_CN) *δ*_H_ 9.88 (1H, s), 9.01 (1H, d, *J* = 7.9 Hz), 8.64 (1H, d, *J* = 7.9 Hz), 8.54 (1H, app. t, *J* = 7.9 Hz), 7.71 (1H, s), 7.40 (1H, s), 7.03 (1H, s), 6.47 (1H, s), 2.23 (3H, s, C*H*_3_), 2.14 (6H, overlapping s, C*H*_3_), 1.92 (3H, s, C*H*_3_) ppm. ^13^C{^1^H} NMR (101 MHz, CDCl_3_) *δ*_C_ 160.1, 157.7, 154.8, 154.4, 149.6, 145.4, 144.6, 143.3, 143.1, 142.7, 141.2, 140.2, 139.9, 138.6, 137.8, 129.3, 128.6, 127.8, 125.1, 124.5, 123.3, 20.9 (*C*H_3_), 20.1 (*C*H_3_), 19.9 (*C*H_3_) ppm. HRMS *m*/*z* found 971.3025; calcd 971.3038 for [C_50_H_40_IrN_10_]^+^. UV-vis *λ*_max_ (MeCN)/nm (*ε*/dm^3^ mol^−1^ cm^−1^) 273 (71 000), 328 (42 000), 376 (39 000).

## Conflicts of interest

There are no conflicts of interest to declare.

## Supplementary Material

RA-011-D1RA07213G-s001

RA-011-D1RA07213G-s002
