# Supplementary material for: Bis-cyclometalated iridium(iii) complexes with terpyridine analogues: syntheses, structures, spectroscopy and computational studies
Source: RSC Adv. 2021 Dec 13;11(63):39718–27. doi: 10.1039/d1ra07213g (PMC9044569; doi:10.1039/d1ra07213g)
Supplement: RA-011-D1RA07213G-s001 [file RA-011-D1RA07213G-s001.pdf]

Electronic Supplementary Material (ESI) for RSC Advances.
This journal is © The Royal Society of Chemistry 2021

Bis-cyclometalated iridium(III) complexes with terpyridine analogues: syntheses, structures, spectroscopy and computational studies

Haleema Y. Otaif, Samuel J. Adams, Peter N. Horton, Simon J. Coles, Joseph M. Beames, Simon J.A. Pope

Electronic Supplementary Information

Contents

**Table S1**	**p2**
**Table S2**	**p3**
**Figure S1**	**p3**
**Figure S2**	**p4**
**Figure S3**	**p5**
**Figure S4**	**p5**
**Table S3**	**p6**
**Table S4**	**p7**
**Table S5**	**p8**
**Table S6**	**p9**
**Table S7**	**p9**
**Table S8**	**p9**
**Figure S5**	**p10**
**Table S9**	**p10**
**Table S10**	**p11**
**Figure S6**	**p11**
**Figure S7**	**p12**

Table S1. Data collection parameters for the crystal structures.

**Compound**	**$[Ir(dpqx1^{-})_2]PF_6$**	**$[Ir(dpqx2^{-})_2]PF_6$**
Formula	$C_{48}F_6H_{37}IrN_{11}OP$	$C_{50.5}ClF_6H_{41}IrN_{10}P$
$D_{calc.}$/ g cm^{-3}	1.701	1.569
μ/mm^{-1}	3.166	6.659
Formula Weight	1121.05	1160.55
Colour	red	red
Shape	prism	plate
Size/mm^3	0.208×0.144×0.024	0.060×0.050×0.010
T/K	100(2)	100(2)
Crystal System	monoclinic	monoclinic
Space Group	$P2_1/n$	$I2/a$
a/Å	15.61910(10)	20.0555(5)
b/Å	17.9773(2)	17.4269(3)
c/Å	15.73360(10)	29.5696(14)
α/°	90	90
β/°	97.8170(10)	108.009(4)
γ/°	90	90
V/Å^3	4376.77(6)	9828.4(6)
Z	4	8
Z'	1	1
Wavelength/Å	0.71075	1.54178
Radiation type	MoK_α	CuK_α
Θ_{min}/°	1.724	2.983
Θ_{max}/°	28.701	68.233
Measured Refl.	85017	60261
Independent Refl.	11289	8958
Reflections with I > 2(I)	9970	6008
R_{int}	0.0371	0.0687
Parameters	569	621
Restraints	0	0
Largest Peak	3.846	2.863
Deepest Hole	-0.784	-1.293
GooF	1.058	1.033
wR_2 (all data)	0.0716	0.1436
wR_2	0.0697	0.1284
R_1 (all data)	0.0356	0.0729
R_1	0.0298	0.0493

Table S2. Selected bond angles for the complexes.

[Ir(**dqxp1**$^-$)$_2$]PF$_6$				[Ir(**dqxp2**$^-$)$_2$]PF$_6$			
**Atom**	**Atom**	**Atom**	**Angle/°**	**Atom**	**Atom**	**Atom**	**Angle/°**
N(1)	Ir(1)	N(2)	77.14(10)	N(1)	Ir(1)	N(4)	76.67(19)
N(1)	Ir(1)	N(32)	110.82(9)	N(1)	Ir(1)	N(31)	174.31(18)
N(31)	Ir(1)	N(1)	170.86(10)	N(1)	Ir(1)	N(34)	104.31(19)
N(31)	Ir(1)	N(2)	108.97(9)	N(4)	Ir(1)	N(34)	92.90(17)
N(31)	Ir(1)	N(32)	76.53(10)	N(31)	Ir(1)	N(4)	108.93(19)
N(2)	Ir(1)	N(32)	89.59(9)	N(31)	Ir(1)	N(34)	76.7(2)
C(45)	Ir(1)	N(1)	90.83(10)	C(1)	Ir(1)	N(1)	81.7(2)
C(45)	Ir(1)	N(31)	82.12(10)	C(1)	Ir(1)	N(4)	158.4(2)
C(45)	Ir(1)	N(2)	93.60(9)	C(1)	Ir(1)	N(31)	92.7(2)
C(45)	Ir(1)	N(32)	158.27(10)	C(1)	Ir(1)	N(34)	93.1(2)
C(15)	Ir(1)	N(1)	82.15(11)	C(1)	Ir(1)	C(31)	90.1(2)
C(15)	Ir(1)	N(31)	91.84(11)	C(31)	Ir(1)	N(1)	96.8(2)
C(15)	Ir(1)	N(2)	159.19(11)	C(31)	Ir(1)	N(4)	91.7(2)
C(15)	Ir(1)	N(32)	95.87(10)	C(31)	Ir(1)	N(31)	82.2(2)
C(15)	Ir(1)	C(45)	88.73(10)	C(31)	Ir(1)	N(34)	158.9(2)

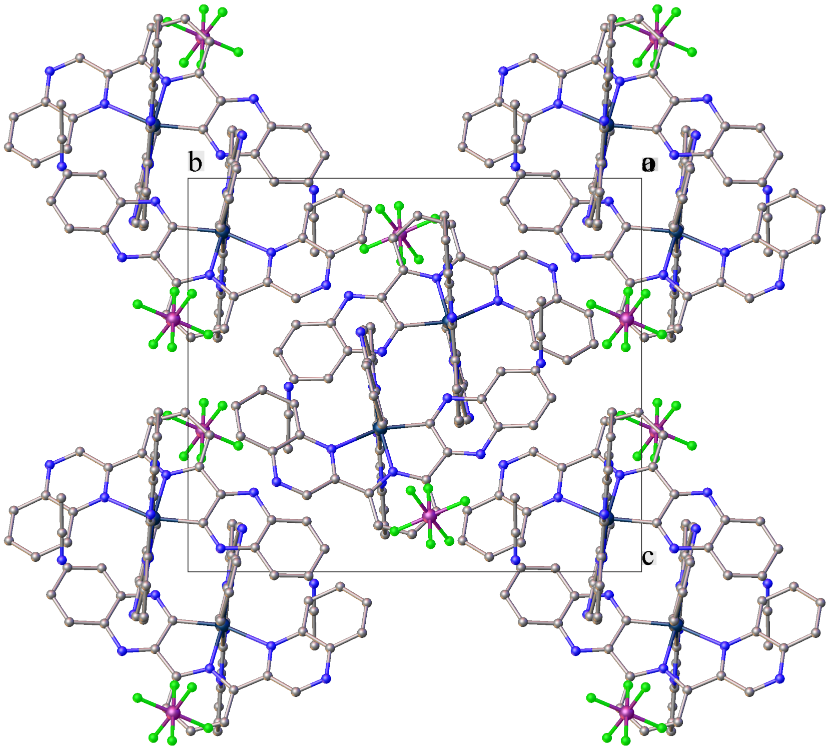

Figure S1. Packing diagram of complex [Ir(**dqxp1**$^-$)$_2$]PF$_6$.

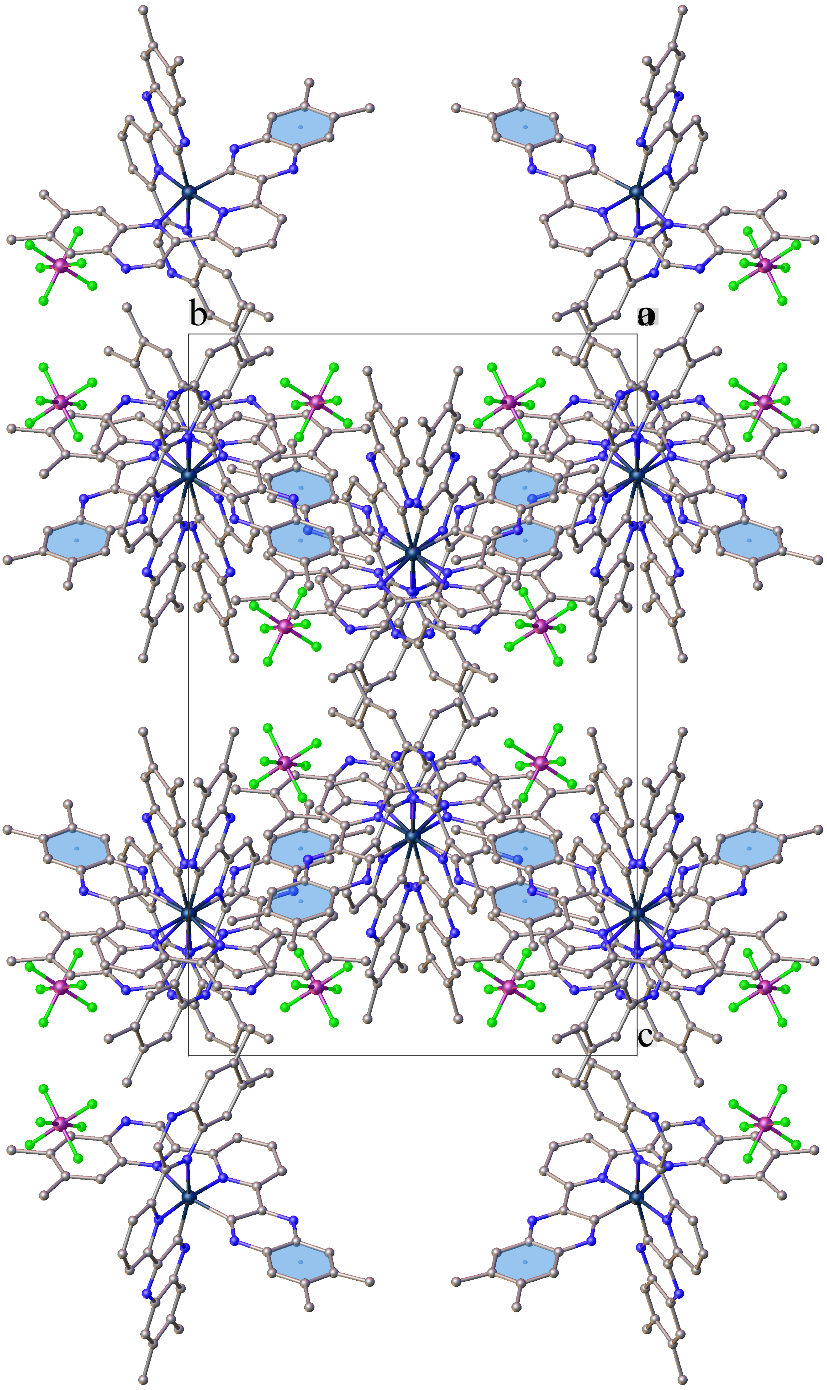

Figure S2. Packing diagram of complex [Ir(**dqxp2**$^{-}$)$_2$]PF_6.

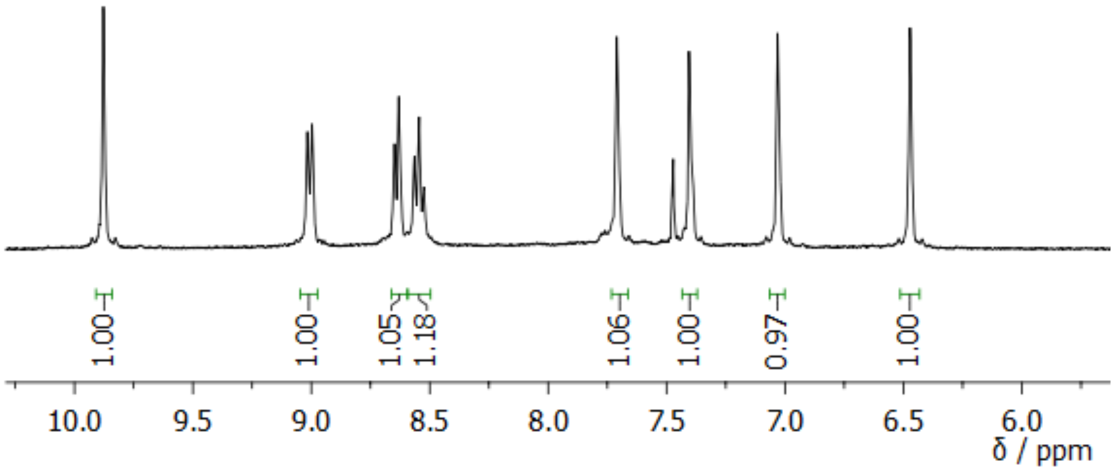

Figure S3. ^{1}H NMR spectrum (CD_3CN) of [Ir(**dqxp2**$^-$)$_2$]PF_6 showing the unique proton environments in the aromatic region.

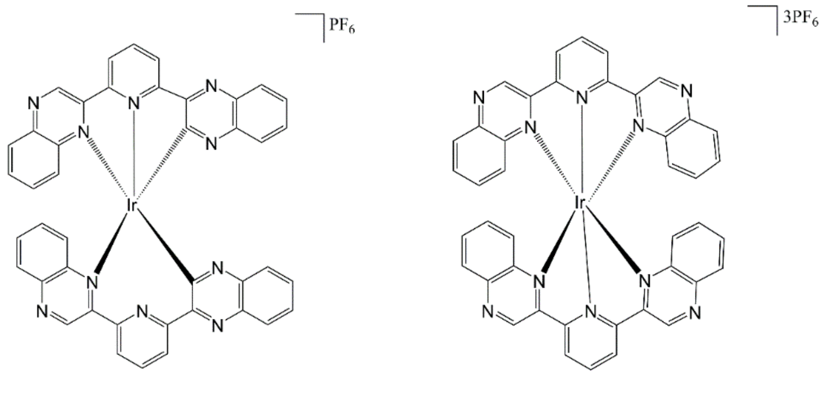

Figure S4. Hypothetical structures comparing the N^N^C and N^N^N homoleptic coordination modes of the tridentate ligands.

Table S3. Calculated energies, bond lengths and angles of the two different coordination modes using B3LYP.

	**N^N^C mode**	**N^N^N mode**
B3LYP Energy	-2266.8188 Hartree<br>-61683.3108 eV	-2267.5832Hartree<br>-61704.11122eV
Internal Energy	-2266.2044 Hartree<br>-61666.5921 eV	-2266.9408 Hartree<br>-61686.6306 eV
ZPE	0.5768 Hartree<br>15.6956 eV	0.6051 Hartree<br>15.6928 eV

**N^N^C mode**		**N^N^N mode**	
Bond	Length (Å)	Bond	Length (Å)
Ir-N_2	2.3227	Ir-N_2	2.169
Ir-N_5	2.0235	Ir-N_5	2.019
Ir-C_{14}	2.0186	Ir-N_{18}	2.158
Ir-C_{40}	2.0014	Ir-N_{41}	2.019
Ir-N_{47}	2.0442	Ir-N_{53}	2.162
Ir-N_{50}	2.2901	Ir-N_{70}	2.159

**N^N^C mode**		**N^N^N mode**	
	Angles (^{o})		Angles (^{o})
N_2-Ir-N_5	75.6969	N_2-Ir-N_5	78.302
C_{14}-Ir-N_5	82.0842	N_2-Ir-N_{70}	88.787
C_{40}-Ir-N_2	98.3608	N_5-Ir-N_{18}	78.455
C_{40}-Ir-N_5	97.8383	N_5-Ir-N_{70}	101.592
C_{14}-Ir-C_{40}	88.4132	N_{18}-Ir-N_{70}	95.677
C_{14}-Ir-C_{40}	88.4132	N_{18}-Ir-N_{53}	88.062
C_{14}-Ir-N_{47}	97.9157	N_{41}-Ir-N_{53}	78.396
C_{14}-Ir-N_{50}	87.3719	N_{41}-Ir-N_{70}	78.453
C_{40}-Ir-N_{47}	82.1621	N_{18}-Ir-N_{41}	101.726
N_{47}-Ir-N_{50}	75.3388	N_{18}-Ir-N_{70}	93.9384

Table S4. Calculated energies, bond lengths and angles of the two different coordination modes using B3LYP/LANL2DZ.

	**N^N^C mode**	**N^N^N mode**
B3LYP Energy	-2266.1273 Hartree<br>-61691.70542 eV	-2267.8926 Hartree<br>-61712.53 eV
Internal Energy	-2266.5124 Hartree<br>-61674.9756 eV	-2267.2498 Hartree<br>-61695.04049 eV
ZPE	0.57674 Hartree<br>15.7112 eV	0.605449 Hartree<br>16.4751 eV

**N^N^C mode**		**N^N^N mode**	
Bond	Length (Å)	Bond	Length (Å)
Ir-N_2	2.030	Ir-N_2	2.164
Ir-N_{27}	2.284	Ir-N_5	2.012
Ir-C_5	2.011	Ir-N_{18}	2.153
Ir-C_{43}	2.011	Ir-N_{41}	2.012
Ir-N_{40}	2.030	Ir-N_{53}	2.158
Ir-N_{61}	2.285	Ir-N_{70}	2.154

**N^N^C mode**		**N^N^N mode**	
	Angles (°)		Angles (°)
N_2-Ir-N_{27}	75.703	N_2-Ir-N_5	78.454
C_5-Ir-N_2	81.887	N_2-Ir-N_{70}	88.496
C_{43}-Ir-N_2	95.535	N_5-Ir-N_{18}	78.604
C_{43}-Ir-N_{27}	91.105	N_5-Ir-N_{70}	101.481
C_5-Ir-C_{43}	91.030	N_{18}-Ir-N_{70}	95.835
C_5-Ir-N_{61}	90.924	N_{18}-Ir-N_{53}	87.838
C_5-Ir-N_{40}	92.944	N_{41}-Ir-N_{53}	78.548
C_{43}-Ir-N_{40}	81.900	N_{41}-Ir-N_{70}	78.605
C_{43}-Ir-N_2	92.621	N_{18}-Ir-N_{41}	101.584
N_{40}-Ir-N_{61}	75.668	N_{18}-Ir-N_{70}	95.835

Table S5. Calculated energies, bond lengths and angles of the two different coordination modes using M062X/LANL2DZ.

	**N^N^C mode**	**N^N^N mode**
M062X Energy	-2266.15195 Hartree<br>-61665.16483 eV	-2266.9041 Hartree<br>-61685.6313 eV
Internal Energy	-2265.53067 Hartree<br>-61648.2587 eV	-2266.2555 Hartree<br>-61667.9822 eV
ZPE	0.584044 Hartree<br>15.8927 eV	0.611566 Hartree<br>16.6416 eV

**N^N^C mode**		**N^N^N mode**	
Bond	Length (Å)	Bond	Length (Å)
Ir-N_2	2.030	Ir-N_2	2.138
Ir-N_{27}	2.297	Ir-N_5	2.004
Ir-C_5	1.976	Ir-N_{18}	2.134
Ir-C_{43}	2.011	Ir-N_{41}	2.004
Ir-N_{40}	2.030	Ir-N_{53}	2.137
Ir-N_{61}	2.298	Ir-N_{70}	2.136

**N^N^C mode**		**N^N^N mode**	
	Angles (°)		Angles (°)
N_2-Ir-N_{27}	75.206	N_2-Ir-N_5	78.789
C_5-Ir-N_2	82.686	N_2-Ir-N_{70}	86.706
C_{43}-Ir-N_2	91.621	N_5-Ir-N_{18}	78.784
C_{43}-Ir-N_{27}	91.829	N_5-Ir-N_{70}	100.989
C_5-Ir-C_{43}	91.008	N_{18}-Ir-N_{70}	97.582
C_5-Ir-N_{61}	91.663	N_{18}-Ir-N_{53}	86.470
C_5-Ir-N_{40}	92.965	N_{41}-Ir-N_{53}	78.773
C_{43}-Ir-N_{40}	82.712	N_{41}-Ir-N_{70}	78.798
C_{43}-Ir-N_2	91.621	N_{18}-Ir-N_{41}	101.398
N_{40}-Ir-N_{61}	75.147	N_{18}-Ir-N_{70}	97.582

Table S6. A comparison of experimentally determined and calculated bond lengths (Å) for the coordination spheres of the complexes.

[Ir(**dqxp1**$^{-}$)$_2$]PF$_6$				[Ir(**dqxp2**$^{-}$)$_2$]PF$_6$			
		**Exp.**	**Calc.**			**Exp.**	**Calc.**
Ir(1)	N(1)	1.999(2)	2.037	Ir(1)	N(1)	2.009(5)	2.036
Ir(1)	N(31)	1.997(2)	2.037	Ir(1)	N(4)	2.191(5)	2.277
Ir(1)	N(2)	2.183(2)	2.279	Ir(1)	N(31)	2.017(5)	2.036
Ir(1)	N(32)	2.199(2)	2.279	Ir(1)	N(34)	2.198(5)	2.276
Ir(1)	C(45)	1.993(3)	2.019	Ir(1)	C(1)	1.999(6)	2.020
Ir(1)	C(15)	1.989(3)	2.019	Ir(1)	C(31)	2.005(7)	2.020

Table S7. The decomposition analysis of the singlet ground state frontier orbitals of [Ir(**dqxp2**$^{-}$)$_2$]$^+$.

Orbital	Moiety Contribution to Orbital (%)						
	Ir 5d	Q1			Q2		
		R1	R2	R3	R1	R2	R3
LUMO +4	1	34	16	0	33	15	0
LUMO +3	1	0	22	27	0	22	27
LUMO +2	1	1	20	29	1	20	29
LUMO +1	4	40	12	0	33	10	0
LUMO	4	31	11	1	39	14	1
HOMO	26	2	3	32	2	3	32
HOMO -1	4	2	4	40	2	5	43
HOMO -2	6	1	5	42	1	5	39
HOMO -3	2	2	4	42	2	4	42
HOMO -4	8	2	1	43	2	1	42

Table S8. The decomposition analysis of the singlet ground state frontier orbitals of [Ir(**dqxp2**$^{-}$)$_2$]$^+$.

[Ir(**dqxp2**$^{-}$)$_2$]PF$_6$		
**Transition**		**Contributing MOs**
1	389.73nm, f= 0.0218	HOMO → LUMO +1 (77%)
2	388.5 nm, f = 0.0162	HOMO → LUMO (79%)
3	352.33 nm, f = 0.0024	HOMO -6 → LUMO +3 (24%)<br>HOMO -4 → LUMO +2 (45%)
4	350.92 nm, f = 0.0003	HOMO -6 → LUMO +2 (11%)<br>HOMO -5 → LUMO +2 (29%)<br>HOMO -4 → LUMO +3 (39%)
5	349.04 nm, f = 0.0311	HOMO -6 → LUMO (27%)<br>HOMO -5 → LUMO (22%)<br>HOMO -3 → LUMO (12%)

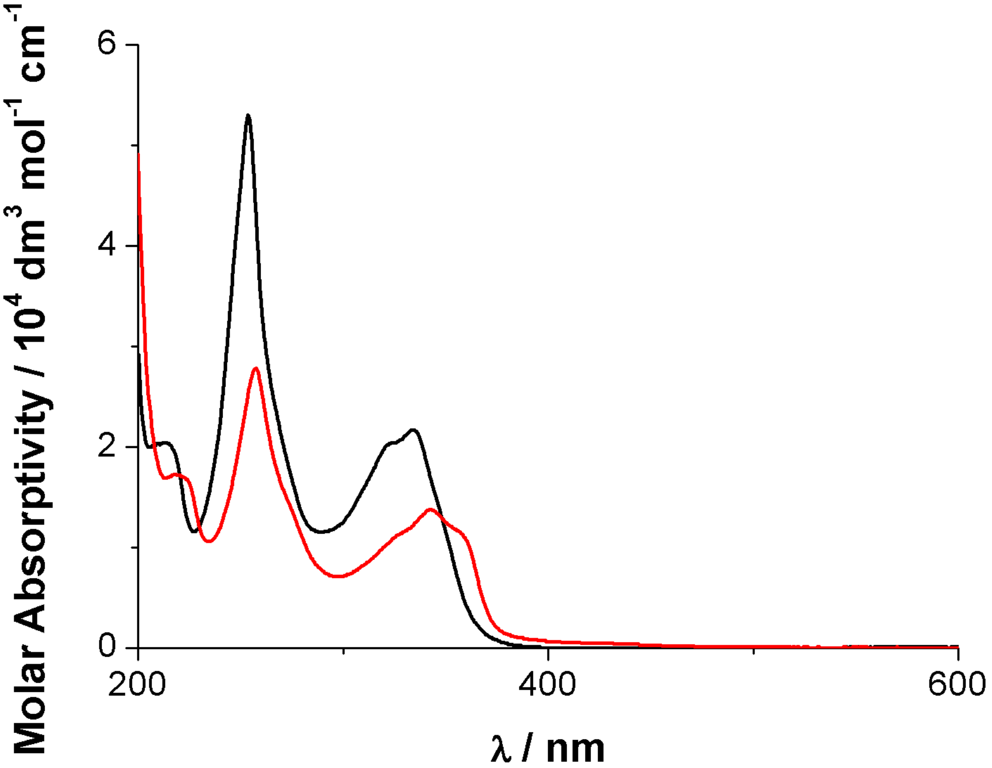

Figure S5. UV-Vis. absorption spectrum of **dqxp1** (black) and **dqxp2** (red) in EtOH solution.

Table S9. Solvatochromic photophysical data for [Ir(**dqxp1**$^-$)$_2$]PF_6.

Solvent	Absorption (ε x 10^4 / $M^{-1}cm^{-1}$) / nm	Emission /nm	Lifetime / ns	Quantum yield (%)
MeCN	260 (9.0), 309 (5.2), 365 (4.5)	614	310	1.8
EtOH	258 (8.1), 318 (5.4), 366 (4.9)	614	198	1.1
MeOH	261 (10.0), 309 (5.5), 365 (5.0)	620	134	0.6

Table S10. Solvatochromic photophysical data for [Ir(**dqxp2**$^-$)$_2$]PF_6.

Solvent	Absorption (ε x 10^4 / $M^{-1}cm^{-1}$) / nm	Emission /nm	Lifetime / ns	Quantum yield (%)
MeCN	265 (7.1), 328 (4.2), 376 (3.9)	617	323	2.0
EtOH	266 (7.7), 330 (4.6), 376 (4.3)	623	209	1.1
MeOH	264 (7.8), 329 (4.5), 376 (4.3)	629	145	0.7

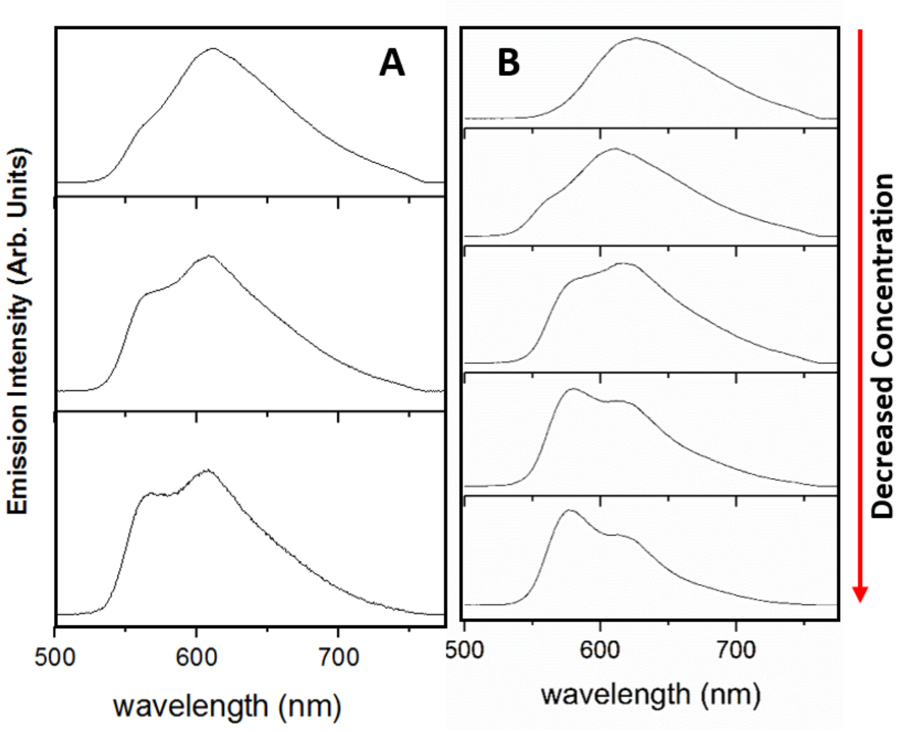

Figure S6. Normalised emission intensities of the two complexes at various dilutions (approx. 10^{-6} to 10^{-3} M), showing a clear spectral response to complex concentration. The left hand panel corresponds to [Ir(**dqxp1**$^-$)$_2$]PF_6, and the right to [Ir(**dqxp2**$^-$)$_2$]PF_6.

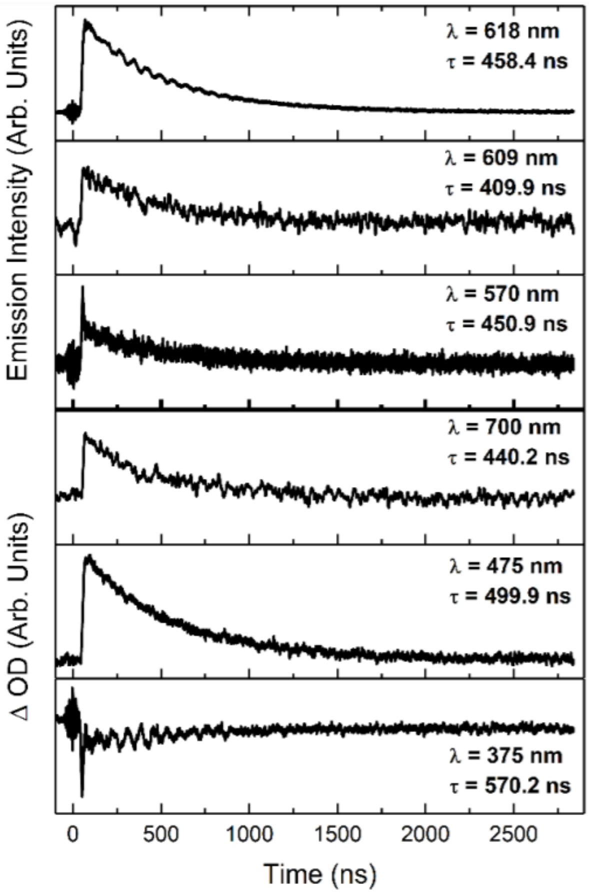

Figure S7. Kinetic traces of the major features of the transient spectra of [Ir(**dqxp1**$^{-}$)$_2$]PF_6 in chloroform at room temperature, aerated. Wavelengths and lifetimes of each trace are inset.
